# Spontaneous deep vein thrombosis in hemophilia A: a case report

**DOI:** 10.4076/1757-1626-2-6390

**Published:** 2009-09-11

**Authors:** Murat Bicer, Murat Yanar, Oktay Tuydes

**Affiliations:** 1 Department of Cardiac Surgery, Kalp Damar Cerrahisi Anabilim Dali Görükle Yerleşkesi, Nilüfer Bursa 16059, Turkey

## Abstract

Venous thromboembolus is an important cause of hospital acquired morbidity and mortality. Venous thrombosis is a very rare occurrence in patients with haemophilia A. The thrombosis originated from the right main and external iliac veins, and effects the cranial segments of the main, deep and superficial femoral veins as an acute phase thrombus. Neither any local anatomic compression nor any predisposing thrombophilic risk factors were identified. We treated the patient with enoxaparine 1 mg/kg twice a day subcutaneously and then started oral anticoagulation with warfarin.

## Introduction

Venous thromboembolus is an important cause of hospital acquired morbidity and mortality [1]. Hemophilia A is a hereditary hemorrhagic disease characterized by deficiency of coagulation Factor VIII. Venous thrombosis has been rarely encountered among patients with hemophilia A [2,3]. Despite the underlying hematological disorders, the development of thrombotic events has been rarely reported in the literature [2,4-7]. In this case report we present the onset of deep vein thrombosis in a 32-year-old male patient with hemophilia A.

## Case presentation

The history of a 32-year-old white male patient that had the diagnosis of hemophilia has revealed two surgeries due to intracapsular hemorrhage of right knee joint and a subdural hematoma. The patient had taken his last Factor VIII replacement 3 months before, and had complaints of pain, edema and warmth on the right lower extremity for two days. On physical examination, his right lower extremity was warmer and 2 cm larger than the left lower extremity in circumference. According to the visual analog scale, the pain score was 6.

**Figure 1:**
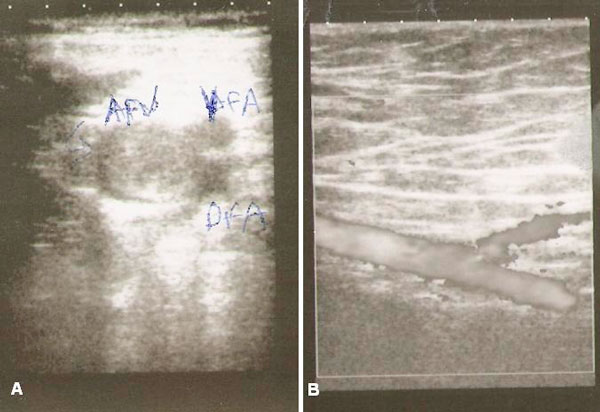
**Doppler ultrasonography showing an acute phase thrombus without any response to augmentation**. **(B)** Restored flow and disappearence of the thrombus after therapy.

At Doppler ultrasonography, originating from the right main and external iliac veins, traversing up to cranial segments of the main, deep and superficial femoral veins an acute phase thrombus was found, without any response to augmentation (Figure 1A). Activated partial thromboplastin time, bleeding time and prothrombin time are given in Table 1. The level of Factor VIII was lower than 2% of normal value.

**Table 1:** Laboratory tests results

Test	Patient's result	Normal value
Platelet count (×10^9^ L^−1^)	320	142.2-424.0
Bleeding time	1 min	1-5 min
PT (s)	15	10-15
PA (%)	73.9	70-130
APTT (sec)	80.1	20-31
INR	1.2	0.9-1.2
Factor VIII level (%)	2	70-150
Protein S (activity; %)	41.9	70-123
Protein C (activity; %)	111.2	70-140
Antithrombin III (%)	97.1	75-125
Fibrinogen (g/L)	6.6	1.8-3.5
Lupus anticoagulant	0.98	1-1.3
Anticardiolipin antibodies	Negative	
Factor V Leiden	Negative	
Prothrombin G20210A	Negative	
MTHFR C677T	Homozygote mutation	
APC resistance	Absent	

PT, prothrombin time; PA, prothrombin activity; APTT, activated partial thromboplastin time; INR, international normalization ratio; APC, activated protein C; MTHFR, methylenetetrahydrofolate reductase.

Patient was treated with enoxaparine 1 mg/kg twice a day and oral warfarin was started 2 days. After achieving an international normalization ratio of 2.0 we stopped giving low molecular weight heparin. Daily coagulation tests and monitorization of thrombocyte levels were performed. The complaints were markedly reduced within the first week of treatment, so the patient was discharged. Doppler ultrasonography was performed at the end of the 6^th^ week and no thrombus was found; a response to augmentation was visible by color Doppler (Figure 1B).

## Discussion

Venous thrombosis is an important cause of hospital acquired morbidity and mortality [1]. Thrombosis is rarely encountered among patients with hemophilia A [2,4]. A total of 12 cases have been reported in the literature, 5 of them regarding deep venous thrombosis [3]. There has been no description of cases with normal coagulation test results and absence of any factor replacement treatment for at least one month which spontaneously developed deep venous thrombosis on the lower extremity.

The most important risk factor for patients with hemophilia A is taking Factor VIII inhibitor bypassing activity (FEIBA) or recombinant activated factor VII (rFVIIa) for inhibitors; however, Factor VII or Factor IX concentrations are also important [3]. Other risk factors can be listed as congenital prothrombotic condition in heterozygote level, deficiency of protein C and Factor V Leiden [8]. In addition to these, Kashyap *et al*. have shown that FII G20210A mutation is another risk factor [8].

Replacement therapy in patients with hemophilia A reduces the thrombophilia development to ratings close to the normal population's [9,10]. Van der Planken reported a deep venous thrombosis development within 18 days after recombinent activated factor VII (rFVIIa) infusion in a 38 years old patient with hemophilia A [11]. Ettingshausen *et al.* described portal venous thrombosis in a patient with the diagnosis of hemophilia A and Factor V G1691A mutation during continuous Factor VIII infusion after jejunal bleeding [6]. Mahmoud *et al.* reported a fatal systemic venous thrombosis resulted from Factor VIIa infusion, which was administered for bleeding control after cardiac surgery [12].

The subjects with methylenetetrahydrofolate reductase (MTHFR) gene polimorphysm have a predisposition to hyperhomocystinemia. In previous studies, a tendency to venous and arterial thrombosis was identified in normal subjects who developed hyperhomocystinemia [13,14]. Although the efficacy of Factor V Leiden was at issue, when it was applied in FII G20210A mutation, the thrombosis formation tendency increased [15]. In this case, because neither a prothrombotic disposition via laboratory tests nor other gene polymorphisms were detected, the presence of homozygote mutation in MTHFR gene made us consider its contribution to the development of thrombosis in our patient. This situation as well suggested that MTHFR gene polymorphism itself may cause a prothrombotic disposition.

The treatment of venous thrombosis in patients diagnosed with thrombotic disorders such as hemophilia A lacks clear information because of the low number of cases. Dargaud *et al.* have used unfractioned heparin for a month subsequent to Factor VIII replacement [2]; Kashyap *et al.* have administered low molecular weight heparin for 9 weeks [8]; Ettinsghausen *et al.* have applied low molecular weight heparin together with Factor VIII replacement, after unfractioned heparin for 14 days. The oral anticoagulant drugs were not a preference since they increase hemorrhage risk.

In our case, the administration of oral anticoagulant drug was initiated after 48 hours, because coagulation tests were normal, Factor V Leiden and prothrombin G20210A mutation were absent, Factor VIII levels were replaced during patient's follow-up, and there was a lack of proteins C and S, which could cause thrombosis in an early phase. This patient was monitored through daily coagulation tests, and marked improvement was observed in his clinical features by color doppler ultrasonography after 6 weeks of treatment. Since these cases are rare, there is no consensus on the therapy; nevertheless, we have been considering oral anticoagulants to be used as treatment, although it requires close monitoring for hemorrhage in these patients.

## Conclusion

Hemophilia A is a hereditary hemorrhagic disorder characterized by Factor VIII's deficiency. Severe Factor VIII deficiency can cause bleeding within the joint, soft tissue and muscle. Arterial and venous thrombosis rarely occur and form a paradox to the underlying disease. Unclearity of the venous thrombosis' mechanism leads to testing alternative treatments. Therefore, it is necessary that the mechanism and treatment of thrombosis be further investigated.

## Abbreviations

FEIBA: factor VIII inhibitor bypassing activity; MTHFR: methylenetetrahydrofolate reductase; rFVIIa: recombinant activated factor VII.

## Consent

Written informed consent was obtained from the patient for publication of this case report and accompanying images. A copy of the written consent is available for review by the journal's Editor-in-Chief.

## Competing interests

The authors declare that they have no competing interests.

## Authors' contributions

MY analyzed and interpreted the patient data and MB and OT contributed in writing the manuscript. All authors read and approved the final manuscript.
